# Effects of combining virtual nature exposure and hypnosis on quality of life in patients with hematologic malignancies undergoing hematopoietic cell transplant: Protocol for an efficacy trial

**DOI:** 10.1016/j.conctc.2026.101706

**Published:** 2026-10-26

**Authors:** Valentyn Fournier, Audrey Laurin, Catherine Paquin, Lynda Rabia, Marine Mandon, Imran Ahmad, Sandie Oberoi, Richard LeBlanc, Jean Roy, David Ogez

**Affiliations:** a Maisonneuve-Rosemont Hospital Research Center, Montréal, Québec, Canada; b Department of Anesthesiology and Pain Medicine, Faculty of Medicine, Université de Montréal, Québec, Canada; c Division of Hematology, Oncology and Cellular Therapy, Hôpital Maisonneuve-Rosemont, Montreal, Quebec, Canada; d Department of Psychology, Faculty of Arts and Sciences, Université de Montréal, Québec, Canada; e Department of Medicine, Université de Montréal, Montreal, Quebec, Canada; f Hematology-Oncology and Cell Therapy University Institute, Hôpital Maisonneuve-Rosemont Research Center, Université de Montréal, Montreal, QC, Canada

**Keywords:** Hematological malignancies, Virtual reality, Hypnosis, Nature exposure, Hematopoietic cell transplant

## Abstract

**Introduction:**

Hematopoietic cell transplantation (HCT) is a key treatment for patients with hematological malignancies, but often results in adverse effects that impact both physical and psychological well-being. This calls for the development of interventions aiming at reducing psychosocial impact of HCT. Among interventions, nature exposure appears promising and efficient via virtual reality. Combined to techniques increasing attention resources allocated to positive elements such as hypnosis, it could represent an innovative solution to preserve quality of life. Following the ORBIT model for the development of non-pharmacological interventions, this study constitutes a Phase IIc efficacy trial. It aims to evaluate the relative efficacy of an intervention combining virtual exposure to nature and hypnosis in patients with a hematological malignancy treated with HCT compared to observation only.

**Methods and analysis:**

The intervention will consist of 2 to 3 weekly 30-min sessions over 2 weeks during aplasia following stem cell infusion, with immersive virtual nature exposure to environments (beach, forest) combined with hypnosis. Eligible patients will be randomly assigned to an intervention or control group. Participants in the intervention group will receive sessions between day +7 and day +21 after HCT. Primary outcome will be the change in quality of life from T1 to T2.

**Perspectives:**

The results of this study will be highly relevant to determine the efficacy of a non-invasive and cost-effective intervention aimed at preserving QoL in patients treated with HCT. Demonstrating its efficacy will pave the way for further development and implementation of a future Phase III randomized controlled trial.

**Ethics and dissemination:**

The project was approved by the Research Ethics Committee of Hôpital Maisonneuve-Rosemont (Project no 2026-4116). Participants will be asked to provide written consent for their participation. Results from this study will be shared through peer-reviewed publications, as well as oral and poster presentations at scientific events. The protocol for this study was preregistered on ISRCTN and raw anonymized data will be available on Open Science Framework (https://osf.io/n598j).

**Trial registration number:**

ISRCTN42049015 (date of registration: November 6th, 2025).

**Protocol version:**

1 (01/11/2025).

**Strengths and limitations of this study**

• This study fills a gap in the literature, proposing a non-pharmacological intervention for patients undergoing hematopoietic cell transplantation
• The intervention is developed following a solid methodological framework
• As a Phase IIc study, it won't explain the processual ways by which this intervention is effective
• This study will inform about the possibility to implement a larger scale randomized controlled trial

## 1 Introduction

Hematopoietic cell transplantation (HCT), whether autologous or allogeneic, is a key treatment for patients with hematological malignancies, but often results in substantial adverse effects that impact both physical and psychological well-being [1], [2]. The initial transplant hospitalization is a particularly critical period. Indeed, following conditioning and HCT, patients go through several weeks of aplasia marked by symptoms such as mucositis, pain, fatigue, and nausea. Those are shown to impact negatively quality of life within two weeks of transplantation, alongside increases in depressive symptoms and symptom burden [3]. Psychological and functional outcomes (e.g., fatigue, insomnia, discomfort, stress) can be attributed to the combined effects of both illness and treatments [4]. These difficulties add to an already heavy psychological burden, as patients with hematological malignancies are among patients with cancer reporting the highest levels of anxiodepressive symptoms and the poorest quality of life [5], [6], [7]. These difficulties are further exacerbated by the protective isolation protocols required during HCT, limiting social contacts, visits, and access to areas outside the hospital room, a source of suffering described by patients [8]. In HCT recipients, there is an urgent need to develop tailored interventions aimed at improving patients’ psychosocial health and quality of life during transplantation [9].

Nature exposure is a promising approach to support well-being. Studies in non-clinical population suggest that regular contact with natural environments is associated with improved psychosocial outcomes. For instance, living near the seaside or frequent access to green spaces has been linked to better physical and psychological health, including lower levels of psychological stress and better mood [10], [11], [12], [13]. Those effects appear to be dose-dependent since they are maximized when people are exposed for over 30 min/week [14]. Other studies have shown that nature exposure reduces rumination on negative experiences, an important determinant of emotional distress [15]. These positive effects of nature exposure can be explained by different theories including the Attention Restoration Theory [13], [16], [17]. According to this theory, natural settings promote psychological recovery by their capacity to slightly captivate attention and offering a sense of spatial expansion, thereby allowing individuals to retrieve their attentional capacities [16]. It is reinforced by experimental results suggesting that nature exposure helps to distract attention away from unpleasant stimuli [18].

Whenever nature exposure is impossible, such as in the context of HCT where patients are immunocompromised and placed in protective isolation for several weeks, alternative solutions are required. Importantly, virtual reality (VR) can bring the benefits of nature in isolation hospital rooms. VR can be defined as simulation of an environment through technological mediation, re-creating sensory and perceptual elements of real-world experiences providing a real immersion in an environment and an experience close to reality [19]. VR is a cost-effective tool for patient care, as it requires minimal staff resources for implementation and frees up time for healthcare professionals [20]. VR has been widely used in general oncology as an educational tool to decrease chemotherapy-related anxiety [21] or decrease cancer-related symptoms [22], [23]. Moreover, virtual nature exposure has been shown to reduce pain, and anxiety in adult oncology [24]. Despite promising literature on the use of VR in oncology, interventions specifically developed for HCT recipients are rare, at an early stage, or not including nature components [25].

VR and techniques bringing more attentional resources to positive elements such as hypnosis may be synergistic to relieve anxiety after HCT. Indeed, exposure to higher nature intensity (that is higher level of vegetation within a landscape) [26] may enhance its effects. Moreover, attention restoration is most likely to occur in calm and immersive environments [17]. Thus, combining both techniques can synergize their effects. Hypnosis, as a state involving focused attention and reduced peripheral awareness, shares core mechanisms with VR. It appears suitable to guide individuals toward guided attentional focus on positive elements of a natural environment, thereby facilitating attentional restoration and improving psychological outcomes. As a therapeutic strategy, the combination of VR and hypnosis has been reported to bring promising results on psychosocial outcomes such as pain, stress, and anxiety in different clinical settings [27], [28].

Based on those elements, our team developed an intervention aiming at bringing the benefits of nature in isolation rooms. This program integrates hypnosis modules which can be used in combination with virtual passive nature exposure using landscapes of forest or seaside. Based on a processual model for development of non-pharmacological interventions (Czajkowski et al., 2015; [29]), the intervention has been preliminary tested in different studies, showing its acceptability and feasibility after some adaptations to the patient experience and context of protective isolation [30].

## 2 Objectives

This aim of this study is to evaluate the relative efficacy of an intervention combining virtual exposure to nature and hypnosis in patients with a hematological malignancy treated with hematopoietic cell transplantation compared to observation only.

## 3 Methods and analysis

This study is led following the SPIRIT 2025 guidelines for elaborating, conducting, and reporting randomized trials [31] (see Supplementary Material 1 for SPIRIT 2025 checklist).

### 3.1 Study design

This study is a randomized, longitudinal, and monocentric project, led at Hôpital Maisonneuve-Rosemont (Montréal, Québec, Canada). This refers to a Phase IIc Efficacy trial according to the ORBIT model [29]. Note that in our institution, both autologous and allogeneic recipients are admitted to the same HCT unit and remain under the same protective isolation measures until neutrophil recovery.

Recruitment of participants will be run from November 1st, 2025, to November 30th, 2026.

### 3.2 Inclusion and exclusion criteria

The target population consists of individuals with a hematological malignancy treated with autologous or allogeneic HCT. Because the two populations may nonetheless differ in length of stay, conditioning intensity, and complications, transplant type will be included as a pre-specified covariate and examined as a potential moderator in exploratory analyses.

The inclusion criteria for both the experimental and the control group are: (a) being over 18 years old, (b) undergoing autologous or allogeneic HCT for a hematologic malignancy, (c) being sufficiently fluent in French to fully understand the recorded hypnosis sessions. The exclusion criteria are: (a) being treated with a HCT at home, (b) having received HCT previously or tandem autologous/allogeneic transplant, (c) having significant cognitive or psychiatric disorders impairing communication with others, or (d) having sensory impairments (deafness, blindness) that impact participation in the study.

### 3.3 Recruitment procedure

A preselection of participants will be conducted based on inclusion and exclusion criteria. Eligible patients will be met by one of the persons responsible for recruitment (CP, MM, or LR) during a pretransplant medical evaluation or their admission to the HCT unit. At this time, patients will have the research project explained and receive the consent form. Patients will be recontacted around half a day later and asked if they have questions. If they agree to participate, they will be asked to sign the consent form. Inclusion and exclusion criteria will then be reviewed by a medical team member to ensure eligibility.

After providing informed consent, participants will be randomly assigned to either the experimental group receiving the intervention or the control group. Allocation will be done following a permutated blocks randomization (blocks size of 4, 6, or 8, randomly fixed) to ensure a global balance and limit predictability of allocation procedure. Allocation list was generated before the beginning of inclusions using the *blockrand* package [32] for R version 4.5.1. Allocation concealment is ensured by keeping sequence secure and only accessible to the person responsible for inclusions. The code for randomization is displayed in Supplementary Material 2.

### 3.4 Intervention

Participants in the experimental group will receive an intervention consisting of virtual exposure sessions to nature environments (forest or beach) combined with hypnosis, each lasting 30 min and delivered over a two-week period. The intervention was developed in collaboration with Super Splendide (www.supersplendide.com), a company specializing in virtual reality application development. It will be delivered via a Meta Quest Pro headset integrating both visual and auditory channels, and includes environments based on 360° audiovisual recording captured in Quebec. The environment includes both visual and auditory components, allowing participants to hear sounds (e.g., waves, wind rustling through leaves) while observing nature.

The virtual nature exposure is accompanied by a pre-recorded hypnotic communication played simultaneously (see Supplementary Material 3 for a transcript of the hypnotic script). This hypnotic guidance aims to direct attention toward the pleasant aspects of the experience. It follows a standardized multi-step structure: (1) a calming induction phase designed to relax the participant and anchor attention in the present experience, (2) progression through the natural environment accompanied by soothing and relaxing verbal suggestions, and (3) gradual reorientation to a fully alert and wakeful state.

Participants in the experimental group will benefit from the intervention starting on Day 7 ± 1 after HCT (T1). This timing was chosen for three reasons. First, the intervention aims to preserve quality of life during the post-infusion period, when the decline is most pronounced [33]. Second, the length of conditioning and the interval between admission and Day 0 vary widely across regimens and type of transplants. Anchoring the intervention at this time standardizes its timing relative to the transplant course across participants. Third, this design provides two pre-intervention assessments, one before conditioning (T0) and one immediately before the first session (T1). During the first session, participants will be introduced to the headset and guided to test the application with a team member (AL). Between Day +7 ± 1 and Day +21 ± 1 after HCT, participants will be invited to complete two to three sessions of virtual nature exposure per week (total of 4 to 6 sessions; maximum a total of 3 h of exposure). Before each session, the team member delivering the intervention will ensure the participant is able to participate. Sessions will be postponed when the clinical state of the participant makes them unable to participate (the participant declines, reports feeling unable to participate). The reason for each postponement or cancellation will be recorded and reported in the final report. The lower threshold is set at 4 sessions to ensure that patients are provided with more than 30 min of exposition weekly.

Outcome measures will be assessed at baseline (before conditioning regimen, T0), after transplantation (Day +7 ± 1) but before the intervention (T1), Day +21 ± 1 after transplantation, after the intervention (T2), at Day +28 ± 2 (T3), and at Day +100 ± 10 (T4) post-transplantation.

This period was chosen because the intervention targets the initial transplant hospitalization, period shared by both populations. Including each population enhances the generalizability of the findings.

The detailed participant timeline describing schedule of enrollment, interventions, and assessments following SPIRIT 2025 guidelines is depicted in Supplementary Material 4 [31]. A schematic graphical representation of the timeline is depicted on Fig. 1.

**Fig. 1:**
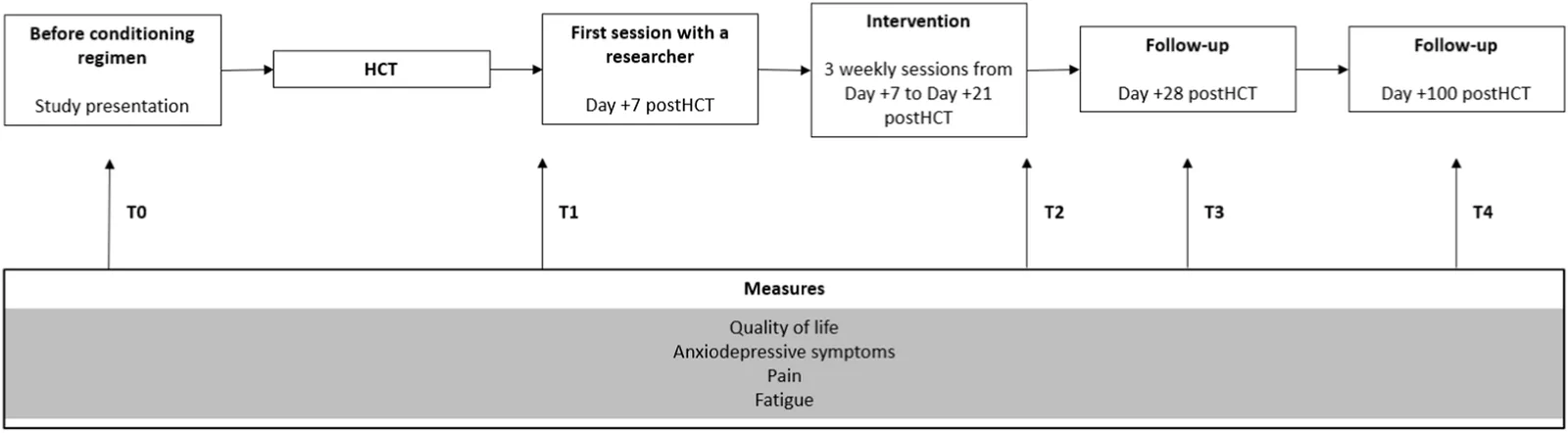
Graphical representation of the procedure Note. HCT: Hematopoietic Cell Transplantation.

### 3.5 Measures

Consent, questionnaire data and sociodemographic information will be provided by participants on a tablet using the software REDCap. Using the platform will limit the risk of missing data by making missing a question not possible.

#### 3.5.1 Primary outcome

Quality of life will be measured using the global score of Functional Assessment of Cancer Therapy – Bone Marrow Transplantation [FACT-BMT] [34], [35]. The FACT-BMT includes four subscales: physical well-being (7 items), social/family well-being (7 items), emotional well-being (6 items) and functional well-being (7 items), complemented by a transplantation-specific module comprising 23 additional items related to symptoms and issues associated with bone marrow transplantation symptoms. Participants are asked to rate each statement on a 5-point Likert scale ranging from 0 (“Not at all”) to 4 (“Very much”). According to the scoring manual, after reversing the indicated items, higher scores reflect better perceived quality of life.

FACT-BMT was selected for three reasons. First, the transplant-specific module captures concerns that are particularly relevant during the inpatient aplasia period. Second, it has been validated in both autologous and allogeneic recipients [35]. Finally, it was the primary outcome of a randomized trial onf a supportive care intervention during HCT hospitalization and was used in a recent pilot randomized trial of a VR psychosocial intervention in the same setting, allowing direct comparison of effect sizes [33], [36].

#### 3.5.2 Secondary outcomes

Anxiodepressive symptoms will be assessed using the Hospital Anxiety and Depression Scale (HADS) [37], [38]. This self-administered instrument comprises 14 items divided into two subscales evaluating depressive symptoms (7 items) and anxiety symptoms (7 items). Each item is rated on a 4-point scale from 0 ("Not at all") to 3 ("Completely agree"). Subscale scores range from 0 to 21, and the global score from 0 to 42, with higher values indicating more severe anxious or depressive symptomatology.

Pain intensity and interference will be measured using the Brief Pain Inventory – Short Form (BPI-SF) [39], [40]. This 9-item self-report questionnaire explores multiple dimensions of pain, including sensory, emotional, and functional aspects. Participants indicate the intensity of their worst, least, average, and current pain on 0-10 numeric rating scales and evaluate the extent to which pain disrupts their daily activities.

Fatigue will be evaluated with the French short version of the Multidimensional Fatigue Inventory – 10 Items [41]. This instrument assesses physical, emotional, and cognitive dimension of fatigue through 10 items rated on a 5-point Likert scale. Higher total score corresponds to greater level of perceived fatigue.

For participants assigned to the experimental condition, potential adverse effects related to virtual reality exposure (i.e., cybersickness) will be evaluated after each session using the Simulator Sickness Questionnaire [42]. This questionnaire comprises 16 items rated on a 4-point Likert scale (from 0 = “Not at all” to 3 = “Severely”) assessing two dimensions of cybersickness: nausea and oculomotor symptoms. Higher score indicates greater symptoms of cybersickness.

Sociodemographic data (i.e., biological sex, gender, age, marital status, level of education) and medical data (hematological diagnosis, transplant conditioning, type of donor (autologous or allogeneic), and relevant HCT physical side effects including fever (≥38.2°C), mucositis (defined as the number of oral/subcutaneous doses of narcotics for mouth pain in the past 24 h), anxiolytic or antidepressant treatments, and acute graft-versus-host disease defined by MAGIC criteria [43] will be collected.

#### 3.5.3 Retention outcomes

Retention will be defined as the proportion of participants who complete the study according to the predefined schedule. Adherence, assessed in the experimental group only, will be defined as the number of sessions completed out of six, as well as the cumulative exposure time. A participant will be considered to have received an adequate dose if they complete at least 4 sessions. Reasons for missed, postponed, or interrupted sessions, as well as reasons for discontinuation or withdrawal from the study, will be systematically recorded. All protocol deviations will be documented.

#### 3.5.4 Power analysis and statistical analysis

Quantitative variables will be summarized as means with their corresponding standard deviation (Mean ± SD) and categorical variables will be presented as frequency and percentages. The evaluation of intervention's effect on outcomes of interest will first rely on univariate analyses, followed by multivariate modeling adapted to the data structure. Only variables demonstrating an association with the outcomes at a significance threshold of *p* < 0.20 in the univariate analysis will be considered in the multivariate model. All inferential analysis will be performed using two-tailed tests, with a significance level equal to 0.05. Missing data will be handled by proceeding to multiple imputation.

An a priori power analysis was conducted using the G*Power 3.1.9.7 software, based on an ANOVA for repeated measures with a between-subject factor, considering quality of life as assessed by FACT-BMT as primary outcome. The effect size *f* was set to 0.25 (medium effect size) [44], [45], the *α* error risk to 0.05, and the *β* factor to 0.20 (power 1- *β* = 0.80). Under these parameters, the required sample size was estimated at n = 86. In accordance with recommendations from the literature [46], an attrition risk should be considered. For this reason, a compensation for potential participant loss should be operated by overrecruiting at Time 1. Based on an anticipated 10% attrition, corresponding to the nonrelapse mortality after allogeneic HCT and drop-out rate for various reasons at the inclusion site, the final target sample size was adjusted to n = 94 participants.

A data monitoring committee will not be established for this study as the trial is considered at minimal risk, involving an intervention with a well-established safety profile, and does not include interim analyses or stopping rules that would require independent oversight. Safety monitoring will be performed by the investigators, with adverse events reviewed at regular intervals. This approach is considered sufficient to ensure participant safety and data integrity.

#### 3.5.5 Feasibility

According to the ORBIT model [47], this study is built on previous team's work. First, a user experience study (Phases Ia and Ib) led in 10 patients showed positive findings on satisfaction and engagement, confirming its relevance [30]. Then, a subsequent feasibility pilot study (Phase IIa and IIb) was led between January and August 2025, providing key element to adapt the intervention. For instance, the study showed that implementation is feasible within protective isolation, while requiring adjustments including supervised sessions of limited time rather than leaving the device in the room [48].

Regarding capacity of recruitment, the study will be led in a transplant unit performing approximately 100 allogeneic HCTs and 40-50 autologous transplants yearly with a survival rate > 95% at 3 months, ensuring enrolment of the target number of patients within a year.

#### 3.5.6 Patient and public involvement

The intervention was developed in direct partnership with patients, ensuring that their perspectives guided both design and implementation. HCT recipients provided critical feedback on users’ experience and acceptability of the intervention. Those were considered within the refinement of the intervention. This collaborative approach ensured that the final version of the application was not only clinically relevant but also patient centered. Finally, the research team includes a patient partner (SO), who is leading the “Espace partenaire”, an organization supported by Hôpital Maisonneuve-Rosemont which aims to include patients in various aspects of care.

#### 3.5.7 Ethics, dissemination, and preregistration

The Research Ethics Committee of Hôpital Maisonneuve-Rosemont (Project no 2026-4116) has approved this project. Any modification of the research protocol will be signaled and submitted to the Research Ethics Committee. Procedure of this study has been preregistered on ISRCTN (Registration number: ISRCTN42049015).

Participants will be informed verbally of the purpose of the research, the course, and the duration of the study. If they declare to be interested, they will be provided with a written consent form. They will be informed they can exercise their right to withdraw from the study at any time of completion, to access and rectify their collected data, and to oppose to the statistical analysis of their data.

The data will be linked to an alphanumeric identification number assigned to each participant by the person responsible for recruitment. A local central registry connecting participants’ identifying information to this identifier will be available and accessible only to the individuals conducting the recruitment, otherwise data from participants will be kept strictly anonymized.

Findings from this study are intended to be disseminated in peer-reviewed specialized professional and scientific journals. In respect to open science principles, supporting materials, raw data, and analysis code will be disseminated on Open Science Framework (OSF) via the following link (https://osf.io/n598j).

## 4 Discussion

Patients with hematological malignancies following HCT are among the most impacted patients with cancer on the psychological and quality of life levels [6], [7]. Among existing non-pharmacological interventions leading to better psychological outcomes, nature exposure has brought promising results. However, protective isolation after HCT does not allow patients to be exposed to a natural environment immediately after transplant. In this context, virtual reality could be a solution to bring nature into isolation rooms. Virtual nature exposure is known to have similar positive results on psychological wellbeing than real nature exposure. Given that effects of nature exposure are dose-dependent and that effects depend on the intensity of nature [14], [26], attentional techniques focusing on bringing more attention resources on positive elements such as hypnosis might maximize those effects. In that sense, this study brings an original perspective to explore the supportive potential of an innovative technology by combining virtual reality and hypnosis. It aims to evaluate the synergistic effect of those two techniques on limiting the impact of hematological malignancies, HCT, and protective isolation on quality of life. Research on this topic being limited, this study prioritizes a methodological sound development of the intervention by being based on a solid model for non-pharmacological interventional research [29], [47]. Because combination of VR and hypnosis is a non-invasive, cost-effective, and easily implementable technology, it holds a great clinical potential for patients with hematological malignancies undergoing stem cell transplant. Gathering results regarding its efficacy could contribute to develop innovative approaches to preserve quality of life in a hardly impacted population. Moreover, this study will pave the way for further studies about the process by which virtual exposure to nature combined with hypnosis work, its long-term effects, and its potential expansion to other oncological conditions.

This study has some limitations. First, it will not inform about the differentiated effects of virtual nature exposure and hypnosis on quality of life. This point would require large-scale studies with distinct conditions. Supplementary studies could also explore the comparative effects of other methods aiming as modulating attentional resources allocated to elements such as mindfulness and acceptance. Moreover, the processual ways by which this intervention is effective should be the object of further fundamental investigation. For instance, suggestibility to hypnosis or sensitivity to beauty of nature could be moderators of the effects of this intervention. Second, session-level suitability criteria were not defined a priori. Although eligibility is assessed before HCT, the decision to postpone or cancel a session relies on participants’ reported ability to participate, which may introduce variability in the delivered dose. Reasons for each deviation will be systematically recorded and will inform the definition of a priori criteria in a future Phase III trial. Third, the intervention starts at Day+7, which coincides with the period of greatest symptom burden after HCT. Although the intervention relies on involuntary attention and requires no active learning, discovering a new device during this period may limit familiarity. An earlier start (around D+0) could be considered in the future. Session-level deferral and tolerance will be reported to inform optimal timing in the next trials. Finally, FACT-BMT was chosen over PROMIS measures, which are now considered as the standard instrument. Our results will therefore not be directly comparable with contemporary registry datasets. A supplementary analysis linking physical function subscale and PROMIS Physical Function metric [49] will partially mitigate this limitation.

In conclusion, the population of patients with hematological malignancies requiring HCT is in great need of interventions aiming at limiting the impact of their illness and its treatment on their quality of life. This study brings an original and innovative way to bring the benefits of nature into hospital rooms and maximize by implementing hypnosis and a technique, leading participants to focus their attention on positive elements. Moreover, it relies on a methodological framework that has led to previous work, ensuring that the intervention is developed in accordance with patients’ reality and institutional constraints. The results of this study will be highly relevant to inform about the efficacy of this intervention on preserving quality of life in patients with hematological malignancies following HCT. Demonstrating its efficacy will pave the way for further studies following the ORBIT model [29], [47]. For instance, implementation of research protocol aiming at deciphering the respective role of each intervention component (e.g., VR alone, hypnosis alone, or a combination of both) could provide a finer understanding of the mechanisms underlying the efficacy of the intervention. Given data are gathered until 100 days after HCT, further studies could also investigate the longer-term effects of the intervention or extend its application over an extended period. Finally, the potential benefits of such an intervention could be tested in other oncological populations and treatment regimens. The larger implementation of the intervention in hematology units will give patients undergoing difficult hospitalization conditions a solution to preserve their well-being.

## CRediT authorship contribution statement

**Valentyn Fournier:** Writing – review & editing, Writing – original draft, Supervision, Project administration, Methodology, Funding acquisition, Conceptualization. **Audrey Laurin:** Writing – review & editing. **Catherine Paquin:** Writing – review & editing. **Lynda Rabia:** Writing – review & editing. **Marine Mandon:** Writing – review & editing. **Imran Ahmad:** Writing – review & editing. **Sandie Oberoi:** Writing – review & editing, Conceptualization. **Richard LeBlanc:** Writing – review & editing. **Jean Roy:** Writing – review & editing, Supervision, Methodology, Funding acquisition, Conceptualization. **David Ogez:** Writing – review & editing, Supervision, Methodology, Funding acquisition, Conceptualization.

## Open science statement

This article is published in open access in accordance with the Creative Commons Attribution Non-Commercial - CC BY-NC 4.0) license. Preregistration form, materials, data, and analysis code supporting this protocol and subsequent study will be available on Open Science Framework (OSF) via the following link (https://osf.io/n598j).

## Funding

This work was supported by the Université de Montréal Maryse and William Brock Chair for clinical research into stem cell transplantation (JR), the Fonds de recherche du Québec – Oncopole (#321941, DO) and the Fonds de recherche du Québec-Santé (salary grant #329980, DO).

## Declaration of competing interest

The authors declare the following financial interests/personal relationships which may be considered as potential competing interests:Jean Roy reports financial support was provided by Université de Montréal Maryse and William Brock Chair for clinical research into stem cell transplantation. David Ogez reports financial support was provided by Fonds de recherche du Québec Oncopole. If there are other authors, they declare that they have no known competing financial interests or personal relationships that could have appeared to influence the work reported in this paper.

## Data Availability

No data was used for the research described in the article.
