# Supplementary material for: Effects of combining virtual nature exposure and hypnosis on quality of life in patients with hematologic malignancies undergoing hematopoietic cell transplant: Protocol for an efficacy trial
Source: Contemp Clin Trials Commun. 2026 Oct 26;54:101706. doi: 10.1016/j.conctc.2026.101706 (PMC13734849; doi:10.1016/j.conctc.2026.101706)
Supplement: Supplementary file 1 [file mmc1.docx]

**Supplementary Material 1.** SPIRIT 2025 Checklist

| **Section / Topic** | **No** | **SPIRIT 2025 checklist item description** | **Reported on page no.** |
| --- | --- | --- | --- |
| **Administrative information** | | |  |
| Title and structured summary | 1a | Title stating the trial design, population, and interventions, with identification as a protocol | 1 |
|  | 1b | Structured summary of trial design and methods, including items from the World Health Organization Trial Registration Data Set | 3 |
| Protocol version | 2 | Version date and identifier | 3 |
| Roles and responsibilities | 3a | Names, affiliations, and roles of protocol contributors | 1 + 14 |
|  | 3b | Name and contact information for the trial sponsor | 1 |
|  | 3c | Role of trial sponsor and funders in design, conduct, analysis, and reporting of trial; including any authority over these activities | 14 |
|  | 3d | Composition, roles, and responsibilities of the coordinating site, steering committee, endpoint adjudication committee, data management team, and other individuals or groups overseeing the trial, if applicable | n.a. |
| **Open science** | | |  |
| Trial registration | 4 | Name of trial registry, identifying number (with URL), and date of registration. If not yet registered, name of intended registry | 3 |
| Protocol and statistical analysis plan | 5 | Where the trial protocol and statistical analysis plan can be accessed | 3 |
| Data sharing | 6 | Where and how the individual de-identified participant data (including data dictionary), statistical code, and any other materials will be accessible | 3 |
| Funding and conflicts of interest | 7a | Sources of funding and other support (e.g., supply of drugs) | 14 |
|  | 7b | Financial and other conflicts of interest for principal investigators and steering committee members | 14 |
| Dissemination policy | 8 | Plans to communicate trial results to participants, healthcare professionals, the public, and other relevant groups (e.g., reporting in trial registry, plain language summary, publication) | 11-12 |
| **Introduction** | | |  |
| Background and rationale | 9a | Scientific background and rationale, including summary of relevant studies (published and unpublished) examining benefits and harms for each intervention | 5-6 |
|  | 9b | Explanation for choice of comparator | 7 |
| Objectives | 10 | Specific objectives related to benefits and harms | 6 |
| **Methods: Patient and public involvement, trial design** | | |  |
| Patient and public involvement | 11 | Details of, or plans for, patient or public involvement in the design, conduct, and reporting of the trial | 11 |
| Trial design | 12 | Description of trial design including type of trial (e.g., parallel group, crossover), allocation ratio, and framework (e.g., superiority, equivalence, non-inferiority, exploratory) | 7-8 |
| **Methods: Participants, interventions, and outcomes** | | |  |
| Trial setting | 13 | Settings (e.g., community, hospital) and locations (e.g., countries, sites) where the trial will be conducted | 7 |
| Eligibility criteria | 14a | Eligibility criteria for participants | 7 |
|  | 14b | If applicable, eligibility criteria for sites and for individuals who will deliver the interventions (e.g., surgeons, physiotherapists) | n.a. |
| Intervention and comparator | 15a | Intervention and comparator with sufficient details to allow replication including how, when, and by whom they will be administered. If relevant, where additional materials describing the intervention and comparator (e.g., intervention manual) can be accessed | 7 |
|  | 15b | Criteria for discontinuing or modifying allocated intervention/comparator for a trial participant (e.g., drug dose change in response to harms, participant request, or improving/worsening disease) | 10 |
|  | 15c | Strategies to improve adherence to intervention/comparator protocols, if applicable, and any procedures for monitoring adherence (e.g., drug tablet return, sessions attended) | 7 |
|  | 15d | Concomitant care that is permitted or prohibited during the trial | 7 |
| Outcomes | 16 | Primary and secondary outcomes, including the specific measurement variable (e.g., systolic blood pressure), analysis metric (e.g., change from baseline, final value, time to event), method of aggregation (e.g., median, proportion), and time point for each outcome | 8-10 |
| Harms | 17 | How harms are defined and will be assessed (e.g., systematically, non-systematically) | 9 |
| Participant timeline | 18 | Time schedule of enrollment, interventions (including any run-ins and washouts), assessments, and visits for participants. A schematic diagram is highly recommended (see Figure) | 7-8  Figure 1 |
| Sample size | 19 | How sample size was determined, including all assumptions supporting the sample size calculation | 10 |
| Recruitment | 20 | Strategies for achieving adequate participant enrollment to reach target sample size | 10 |
| **Methods: Assignment of interventions** | | |  |
| Randomization: |  |  |  |
| Sequence generation | 21a | Who will generate the random allocation sequence and the method used | 7 |
|  | 21b | Type of randomization (simple or restricted) and details of any factors for stratification. To reduce predictability of a random sequence, other details of any planned restriction (e.g., blocking) should be provided in a separate document that is unavailable to those who enroll participants or assign interventions | 7 |
| Allocation concealment  mechanism | 22 | Mechanism used to implement the random allocation sequence (e.g., central computer/telephone; sequentially numbered, opaque, sealed containers), describing any steps to conceal the sequence until interventions are assigned | 7 |
| Implementation | 23 | Whether the personnel who will enroll and those who will assign participants to the interventions will have access to the random allocation sequence | 7 |
| Blinding | 24a | Who will be blinded after assignment to interventions (e.g., participants, care providers, outcome assessors, data analysts) | 7 |
|  | 24b | If blinded, how blinding will be achieved and description of the similarity of interventions | n.a. |
|  | 24c | If blinded, circumstances under which unblinding is permissible, and procedure for revealing a participant’s allocated intervention during the trial | n.a. |
| **Methods: Data collection, management, and analysis** | | |  |
| Data collection methods | 25a | Plans for assessment and collection of trial data, including any related processes to promote data quality (e.g., duplicate measurements, training of assessors) and a description of trial instruments (e.g., questionnaires, laboratory tests) along with their reliability and validity, if known. Reference to where data collection forms can be accessed, if not in the protocol | 8 |
|  | 25b | Plans to promote participant retention and complete follow-up, including list of any outcome data to be collected for participants who discontinue or deviate from intervention protocols | 10 |
| Data management | 26 | Plans for data entry, coding, security, and storage, including any related processes to promote data quality (e.g., double data entry; range checks for data values). Reference to where details of data management procedures can be accessed, if not in the protocol | n.a. |
| Statistical methods | 27a | Statistical methods used to compare groups for primary and secondary outcomes, including harms | 10 |
|  | 27b | Definition of who will be included in each analysis (e.g., all randomized participants), and in which group | 10 |
|  | 27c | How missing data will be handled in the analysis | 10 |
|  | 27d | Methods for any additional analyses (e.g., subgroup and sensitivity analyses) | 10 |
| **Methods: Monitoring** | | |  |
| Data monitoring committee | 28a | Composition of data monitoring committee (DMC); summary of its role and reporting structure; statement of whether it is independent from the sponsor and funder; conflicts of interest and reference to where further details about its charter can be found, if not in the protocol. Alternatively, an explanation of why a DMC is not needed | 10 |
|  | 28b | Explanation of any interim analyses and stopping guidelines, including who will have access to these interim results and make the final decision to terminate the trial | n.a. |
| Trial monitoring | 29 | Frequency and procedures for monitoring trial conduct. If there is no monitoring, give explanation | 10 |
| **Ethics** | | |  |
| Research ethics approval | 30 | Plans for seeking research ethics committee/institutional review board approval | 11 |
| Protocol amendments | 31 | Plans for communicating important protocol modifications to relevant parties | 11-12 |
| Consent or assent | 32a | Who will obtain informed consent or assent from potential trial participants or authorized proxies, and how | 7 |
|  | 32b | Additional consent provisions for collection and use of participant data and biological specimens in ancillary studies, if applicable | n.a. |
| Confidentiality | 33 | How personal information about potential and enrolled participants will be collected, shared, and maintained in order to protect confidentiality before, during, and after the trial | 7 |
| Ancillary and post-trial care | 34 | Provisions, if any, for ancillary and post-trial care, and for compensation to those who suffer harm from trial participation | n.a. |

Citation: Chan A-W, Boutron I, Hopewell S, Moher D, Schulz KF, et al. SPIRIT 2025 statement: updated guideline for protocols of randomised trials. BMJ 2025;389:e081477. <https://dx.doi.org/10.1136/bmj-2024-081477>

© 2025 Chan A-W et al. This is an Open Access article distributed under the terms of the Creative Commons Attribution License (<https://creativecommons.org/licenses/by/4.0/>), which permits unrestricted use, distribution, and reproduction in any medium, provided the original work is properly cited.

**Supplementary Material 2.** Randomization code

library(blockrand)

set.seed(2025)

n_total <- 96

arms <- c("Controle", "Experimental")

rand <- blockrand(n = n_total,

num.levels = length(arms),

levels = arms,

block.sizes = c(4,6,8))

rand$ID <- 1:n_total

rand_list <- rand[, c("ID", "treatment", "block")]

write.csv(rand_list, "liste_randomisation.csv", row.names = FALSE)

**Supplementary Material 3.** Hypnotic script (Translation from French to English)

Forest Script

You may settle in comfortably, make yourself at ease.

Good. I invite you to take a deep breath, and then exhale deeply, very deeply.

Excellent.

Now you can allow your body to relax, to release tension, with each inhalation and exhalation.

Let yourself be guided by your breathing, as if you were descending deeply into a state of relaxation, a state of deep calm. You can even allow your imagination to imagine bubbles of oxygen circulating around you. You can inhale... and exhale. The idea is simply to let this relaxation settle in as comfortably as possible. Very good.

With each inhalation... exhalation... your body relaxes more deeply, as if you were allowing it to rest there, on the chair or the bed... comfortably.

As you allow yourself to go deeper into this state of deep relaxation... very deep... surrounded by pleasant, comfortable oxygen, your body settles into a pleasant calm, resting comfortably on the chair. You can allow yourself to forget this body, to let it rest quietly. Make no effort to relax—do nothing. There is no need to use your energy for that; keep it for later... excellent. And if you feel any discomfort, simply notice it but make no effort to change it. Let the relaxation do it for you—let relaxation bring you comfort.

And your imagination can go even deeper into this state of comfort... with each inhalation, exhalation. You can allow yourself to enter as deeply as possible into this experience of relaxation. And in a few moments, simply let your imagination settle pleasantly in a place of comfort and relaxation.

You can now imagine yourself comfortably in the forest.

Simply look around you—look at the sky, the landscape, the trees... observe the place you are in. Look to the right, to the left... Excellent.

Your body continues to relax while your eyes can see, and your ears can hear. Perhaps you can hear the song of the wind... through the trees, around you, comfortably, allowing you to be pleasantly immersed in this experience—relaxed. And as you listen closely, let yourself be soothed by the birdsong, connecting with these sounds that bring calm—still relaxed, still at ease.

And while you are in this place, simply notice how your body connects to this relaxation—how settled your body feels—connected to nature, doing nothing, simply immersed in these sounds... these images... Some people may even sense the temperature of the place they are in; with imagination, one can be even more immersed, surrounded by this natural warmth or coolness.

There are some people—I don’t know if you are one of them—who can even perceive the scent of pine, the fragrance of flowers... in this calm and peaceful forest.

Contemplate nature, the calmness, what soothes you. Excellent.

While you are immersed in this experience, you can allow your body to rest, as if it too could be in nature—relaxed, comfortable... as if there were a connection between what the mind sees and how the body responds. Excellent.

And now, I invite your body to repair any discomforts... During this time, do nothing... simply allow the experience to guide you through this moment of relaxation and comfort. Very good.

And perhaps this experience, this place, bring to mind another moment you’ve already lived... in nature, effortlessly... You can reconnect with pleasant moments from your life—positive, comfortable memories... remembering times of relaxation, joy, and peace, as you wish.

And to allow you to fully enjoy this moment and let your body feel what the mind suggests, I will remain silent for three minutes, so that you may simply experience it in your own way, comfortably.

Very good. Let yourself be guided by these calming images. Notice how this peacefulness relaxes you—like being by a warm fire... in a clearing... Relaxed.

Excellent. If you wish, during this experience, it is possible to record the benefits of this journey into nature—the benefits for your body as it relaxes. You can even anchor this feeling. To do so, make a simple gesture: touch the thumb and index finger of one hand, right or left—whichever you prefer.

Touching the thumb and index finger of one hand, right or left, is a way to record your relaxed, comfortable state—as if anchoring this experience within your body.

The body is more comfortable, the mind is freer, enjoying the present moment, relaxed. And the next time you make this gesture—touching the thumb and index finger of this hand—it will be a signal allowing you to return, even without today’s images, to this state of comfort you are in now. Very good.

The next time you make this gesture—touching the thumb and index finger—it will signal that you can easily return to this pleasant sensation in your body. You will be able to recreate this pleasant feeling at any time you wish. Excellent.

Gradually now... you can begin to prepare to leave this experience, to move away from this place.

Let yourself be guided by the music, which now begins to bring you energy, gradually restoring vitality.

I now invite you to focus on your breathing.

Take a deep breath. Simply reconnect with your breathing. See the oxygen circulating around you.

As this oxygen moves comfortably around you, you can begin to feel energy returning to your body. This oxygen provides all the energy you need to regain movement.

And don’t be surprised if, even as your energy returns, you continue to feel relaxed and at ease. Let yourself be guided by this music, by these bubbles of oxygen, and you can begin to rise gradually to full wakefulness, emerging from this experience full of energy, with a comfortable body and a clear mind.

At your own pace, begin taking deep inhalations and exhalations… That’s it… regaining all the energy you need. When you are ready—when you feel your comfortable body again, the contact of your feet on the floor, the support of the chair—you will regain awareness of all the sensations of the room, its temperature, its sounds.

Once you have recovered all these sensations and are ready to emerge from this experience, simply indicate it by raising your hand, at your own pace.

Excellent. Thank you very much.

Beach Script

Are you comfortable? At ease?

Good. I will simply ask you to take a deep breath, and then exhale deeply, very deeply.

Excellent.

Now, I invite you to let your body relax, to unwind on this chair, with each inhalation and exhalation.

Let yourself be guided by your breathing, as if you were descending deeply into a state of relaxation, a state of deep calm. You can even allow your imagination to imagine bubbles of oxygen circulating around you. You can inhale... and exhale. The idea is simply to allow this relaxation to settle as comfortably as possible. Very good.

With each inhalation... exhalation... your body relaxes more deeply, as if you were allowing it to rest there, on the chair or bed... comfortably.

As you go deeper into this state of deep relaxation... very deep... surrounded by pleasant, comfortable oxygen, your body settles pleasantly into relaxation, resting comfortably. You can allow yourself to forget this body, to let it rest quietly. Make no effort to relax—do nothing. There is no need to use your energy for that; keep it for later... excellent. And if you feel any discomfort, simply note it without trying to change it. Let relaxation take care of it for you.

And your imagination can go even deeper into this state of comfort... with each inhalation, exhalation. In a few moments, simply allow your imagination to settle pleasantly in a place of rest and relaxation.

You can now imagine yourself comfortably on the beach.

Simply look around you, notice where you are—look to the right, to the left—and hear the pleasant sound of the wind.

Perhaps you can hear the ocean... and the waves around you, comfortably, allowing yourself to be completely immersed in this pleasant, relaxing experience. Your body continues to relax as your eyes can see, your ears can hear—perhaps even the song of the seabirds—connecting with these sounds that bring calm and serenity.

And while you are in this place, notice how your body connects with this relaxation—how grounded it feels—as you face the sea, doing nothing, simply immersed in these sounds... these images... Some people may even feel the cool breeze, the temperature of this place; with imagination, you can become even more immersed in this sensation.

There are some people—I don’t know if you are one of them—who can smell the salty air of the sea... of this beach... or perhaps another one.

To contemplate nature, the calm, what soothes you. Excellent.

While you are immersed in this experience, you can allow your body to rest, as if it too could be on this beach—relaxed, comfortable... as if there were a connection between what the mind sees and how the body reacts. Excellent.

And now, I invite your body to repair any discomforts... During this time, do nothing... simply allow the experience to guide you through this moment of relaxation and comfort. Very good.

And perhaps this experience, this place, bring to mind another moment you have lived... at the beach, effortlessly... You can reconnect with pleasant moments from your life—positive, comfortable memories... remembering times of relaxation, joy, and peace, as you wish.

And to allow you to fully enjoy this moment and let your body feel what the mind suggests, I will remain silent for three minutes, so that you may simply experience it in your own way, comfortably.

Very good. Let yourself be guided by these soothing images. Notice how the setting sun relaxes you—like being by a fire... at the beach... Relaxed.

Excellent. If you wish, during this experience, it is possible to record the benefits of this journey at the beach—the benefits for your body as it relaxes. You can even anchor this sensation. To do so, make a simple gesture: touch the thumb and index finger of one hand, right or left—whichever you prefer.

Touching the thumb and index finger of one hand, right or left, is a way to record your relaxed, comfortable state—as if anchoring this experience within your body.

The body is more comfortable, the mind is freer, enjoying the present moment, relaxed. And the next time you make this gesture—touching the thumb and index finger of this hand—it will be a signal allowing you to return, even without today’s images, to this state of comfort you are in now. Very good.

The next time you make this gesture—touching the thumb and index finger—it will signal that you can easily return to this pleasant sensation in your body. You will be able to recreate this pleasant feeling at any time you wish. Excellent.

Gradually now... you can begin to prepare to leave this experience, to move away from this beach.

Let yourself be guided by the music, which now begins to bring you energy, gradually restoring vitality.

I now invite you to focus on your breathing.

Take a deep breath. Simply reconnect with your breathing. See the oxygen circulating around you.

As this oxygen moves comfortably around you, you can begin to feel energy returning to your body. This oxygen provides all the energy you need to regain movement.

And don’t be surprised if, even as your energy returns, you continue to feel relaxed and at ease. Let yourself be guided by this music, by these bubbles of oxygen, and you can begin to rise gradually to full wakefulness, emerging from this experience full of energy, with a comfortable body and a clear mind.

At your own pace, begin taking deep inhalations and exhalations… That’s it… regaining all the energy you need. When you are ready—when you feel your comfortable body again, the contact of your feet on the floor, the support of the chair—you will regain awareness of all the sensations of the room, its temperature, its sounds.

Once you have recovered all these sensations and are ready to emerge from this experience, simply indicate it by raising your hand, at your own pace.

Excellent. Thank you very much.

**Supplementary Material 4.** SPIRIT 2025 Participant timeline, Schedule of enrollment, interventions, and assessments

|  | **Trial period** | | | | | | | | | | |
| --- | --- | --- | --- | --- | --- | --- | --- | --- | --- | --- | --- |
|  | Enrollment | | | Post-randomization | | | | | | Follow-up | |
| **Timepoint** | *-t_i_* | 0 | T0  Before conditioning | T1  Day +7±1  Session 1 | Session 2 | Session 3 | Session 4 | Session 5 | T2  Day +21±1  Session 6 | T3  Day +28±2 | T4  Day +100±10 |
| **Enrollment** | | | | | | | | | | | |
| Eligibility screen | x |  |  |  |  |  |  |  |  |  |  |
| Informed consent | x |  |  |  |  |  |  |  |  |  |  |
| Randomization |  | x |  |  |  |  |  |  |  |  |  |
| **Intervention group** | | | | | | | | | | | |
| Intervention |  |  |  | x | x | x | x | x | x |  |  |
| SSQ assessment |  |  |  | x | x | x | x | x | x |  |  |
| **Assessments (both groups)** | | | | | | | | | | | |
| Sociodemographic data |  | x |  |  |  |  |  |  |  |  |  |
| Diagnosis, transplant conditioning, type of donor |  | x |  |  |  |  |  |  |  |  |  |
| FACT-BMT |  |  | x | x |  |  |  |  | x | x | x |
| HADS |  |  | x | x |  |  |  |  | x | x | x |
| BPI-SF |  |  | x | x |  |  |  |  | x | x | x |
| MFI |  |  | x | x |  |  |  |  | x | x | x |
| HCT side effects (when applicable) |  |  |  | x |  |  |  |  |  |  | x |
| Acute GVHD (when applicable) |  |  |  | x |  |  |  |  |  |  | x |

*Note.* SSQ: Simulator Sickness Questionnaire; FACT-BMT: Functional Assessment of Cancer Therapy-Bone Marrow Transplant; HADS: Hospital Anxiety Depression Scale; BPI-SF: Brief Pain Inventory-Short Form; MFI: Multidimensional Fatigue Inventory; HCT: Hematopoietic Cell Transplant; GVHD: Graft-Versus-Host Disease. Discontinued lines indicate continuous assessment when event occurs.
